# Supplementary material for: Differences in Work and Commuting Accidents between Employees and Students at Higher Education Institutions in Rhineland-Palatinate, Germany, from December 2014 to December 2019
Source: Int J Environ Res Public Health. 2023 Jan 30;20(3):2462. doi: 10.3390/ijerph20032462 (PMC9915262; doi:10.3390/ijerph20032462)
Supplement: Supplementary file 1 [file ijerph-20-02462-s001.zip › ijerph-2069649-supplementary.pdf]

Article

Differences in work and commuting accidents between employees and students at higher education institutions in Rhineland-Palatinate, Germany, from December 2014 to December 2019

Supplementary Tables

Table S1: Total number of students enrolled in the winter terms of academic years 2014–2015 to 2019–2020.

Higher Education Institution	2014–2015	2015–2016	2016–2017	2017–2018	2018–2019	2019–2020
German University of Administrative Sciences Speyer	371	402	430	343	353	343
University of Applied Sciences Kaiserslautern	5,828	5,960	6,109	6,216	6,107	6,271
University of Applied Sciences Trier	8,006	7,919	7,845	7,555	7,357	7,115
University of Koblenz	8,925	8,970	9,176	9,713	9,837	9,788
University of Applied Sciences Mainz	5,116	5,194	5,356	5,462	5,607	5,630
University of Ludwigshafen	4,293	4,368	4,320	4,478	4,674	4,596
University of Worms	3,362	3,437	3,555	3,670	3,722	3,747
Technical University Kaiserslautern	14,034	14,242	14,418	14,668	14,612	14,427
Catholic University of Mainz	1,239	1,262	1,297	1,409	1,438	1,484
Technical University Bingen	2,628	2,627	2,641	2,708	2,731	2,559
University of Koblenz-Landau	15,576	15,757	16,483	16,981	17,856	17,949
University of Trier	14,306	13,751	13,210	12,714	12,475	12,293
Johannes Gutenberg University Mainz	34,951	33,132	32,573	31,819	31,588	31,544

Table S2: Total number of employees from 2014 to 2019.

Higher Education Institution	2014	2015	2016	2017	2018	2019
German University of Administrative Sciences Speyer	232	229	242	235	248	237
University of Applied Sciences Kaiserslautern	699	742	724	724	760	788
University of Applied Sciences Trier	941	956	951	961	984	977
University of Koblenz	834	893	958	976	956	979
University of Applied Sciences Mainz	755	742	762	825	862	829
University of Ludwigshafen	555	612	653	601	616	637
University of Worms	351	366	380	385	383	391
Technical University Kaiserslautern	3,000	3,056	3,393	3,391	3,470	3,530
Catholic University of Mainz	182	182	187	219	201	216
Technical University Bingen	267	288	308	312	337	330
University of Koblenz-Landau	1,705	1,781	1,945	1,939	2,104	2,332
University of Trier	2,153	2,208	2,175	2,187	1,870	1,845
Johannes Gutenberg University Mainz	13,670	13,927	13,759	13,390	13,755	13,925

Table S3. Calculation of the modified thousand-men quotas (mTMQs) of employees and students for the different institutions from December 2014 to December 2019. The mTMQ describes the rate of injuries per 1,000 employees or 1,000 students by adding up the total number of accidents during this period, multiplying this by 1,000, and then dividing the result by the average number of employees or students at the institution during the five-year period.

Institution	Mean number of employees, December 2014 to December 2019	Mean number of accidents (employees), December 2014 to December 2019	Modified thousand-men quotas of employees	Mean number of students, December 2014 to December 2019	Mean number of accidents (students), December 2014 to December 2019	Modified thousand-men quotas of students
German University of Administrative Sciences Speyer	237.5	2.8	11.79	377	1.2	3.18

**University of Applied Sciences Kaiserslautern**	738.7	3	4.06	6,088.3	17.6	2.89
**University of Applied Sciences Trier**	962.2	4	4.16	7,647.3	27	3.53
**University of Koblenz**	937.9	1	1.07	9,410.5	31.8	3.38
**University of Applied Sciences Mainz**	796.6	1.6	2.01	5,398.4	14.2	2.63
**University of Ludwigshafen**	615.6	1	1.62	4,456.9	17.2	3.86
**University of Worms**	377	0.8	2.12	3,587.7	9	2.51
**Technical University Kaiserslautern**	3,315	12.6	3.8	1,4434.1	83.8	5.81
**Catholic University of Mainz**	197.6	0.4	2.02	1,353.5	6.8	5.02
**Technical University Bingen**	308.7	1	3.24	2,660.1	11	4.14
**University of Koblenz-Landau**	1,957.5	3	1.53	16,767.9	137.6	8.2
**University of Trier**	2,087.8	2.4	1.15	13,089.9	46.4	3.54
**Johannes Gutenberg University Mainz**	13,725.7	27	1.97	32,454.5	162.8	5.02
